# Nanoscale organization of Nicastrin, the substrate receptor of the γ-secretase complex, as independent molecular domains

**DOI:** 10.1186/s13041-021-00855-x

**Published:** 2021-10-13

**Authors:** Shekhar Kedia, Kousik Mandal, Pallavi Rao Netrakanti, Mini Jose, Sangram S. Sisodia, Deepak Nair

**Affiliations:** 1 Centre for Neuroscience, Indian Institute of Science, 560012 Bangalore, India; 2 Centre for Molecular Neurobiology, Department of Neurobiology, The University of Chicago, 60637 Chicago, IL USA

**Keywords:** Nicastrin, Synapse, Alzheimer’s disease, Presenilin, APP, Super resolution microscopy, STED, Secretase

## Abstract

Alterations in the canonical processing of Amyloid Precursor Protein generate proteoforms that contribute to the onset of Alzheimer’s Disease. Modified composition of γ-secretase or mutations in its subunits has been directly linked to altered generation of Amyloid beta. Despite biochemical evidence about the role of γ-secretase in the generation of APP, the molecular origin of how spatial heterogeneity in the generation of proteoforms arises is not well understood. Here, we evaluated the localization of Nicastrin, a γ-secretase subunit, at nanometer sized functional zones of the synapse. With the help of super resolution microscopy, we confirm that Nicastrin is organized into nanodomains of high molecular density within an excitatory synapse. A similar nanoorganization was also observed for APP and the catalytic subunit of γ-secretase, Presenilin 1, that were discretely associated with Nicastrin nanodomains. Though Nicastrin is a functional subunit of γ-secretase, the Nicastrin and Presenilin1 nanodomains were either colocalized or localized independent of each other. The Nicastrin and Presenilin domains highlight a potential independent regulation of these molecules different from their canonical secretase function. The collisions between secretases and substrate molecules decide the probability and rate of product formation for transmembrane proteolysis. Our observations of secretase nanodomains indicate a spatial difference in the confinement of substrate and secretases, affecting the local probability of product formation by increasing their molecular availability, resulting in differential generation of proteoforms even within single synapses.

**Supplementary Information:**

The online version contains supplementary material available at 10.1186/s13041-021-00855-x.

## Background

γ-secretases are the multimeric enzyme complexes consisting of four subunits, namely, Presenilin 1 (PS1), Nicastrin (NCT), Anterior Pharynx Defective 1 (APH1) and Presenilin Enhancer 2 (PEN2) [1]. NCT is a type 1 transmembrane glycoprotein and is the largest subunit of the γ-secretase enzyme complex. It is essential for the cleavage of several γ-secretase substrate molecules, importantly for Notch receptors and Amyloid Precursor Protein (APP) [1, 2]. NCT is necessary for the final processing of APP through either the non-amyloidogenic or the amyloidogenic pathway [3]. The APP processing by γ-secretase through the amyloidogenic pathway results in the generation of Amyloid beta (Aβ), often linked with the pathological progression of the Alzheimer’s disease (AD) [4]. AD is the most prevalent form of dementia in the elderly. It has been already shown that APP cleavage is impaired with inhibition of NCT function in vitro and in vivo [5]. It pinpoints to an indispensable role of NCT as a functional component of γ-secretase for regulated intramembrane proteolysis of APP. Emerging studies suggest that NCT plays a critical role in the assembly of the γ-secretase complex and in the docking of the substrate molecules to the catalytic zone [5, 6]. NCT has also been identified as a substrate selector molecule that allows substrates that have shedded their ectodomain to be cleaved by the catalytic subunit of the γ-secretase. NCT is also known to interact with various proteoforms of APP and several other substrate molecules such as Notch [6–10]. It is already known that alterations in the balance of the proteoforms generated through the non-amyloidogenic and amyloidogenic pathway are crucial contributors to the development and progression of AD [11].

The spatial proximity of γ-secretase to its substrate either by local confinement within nanodomains or by random diffusional collisions on the plasma membrane is a requisite for generation of different proteoforms [12–15]. Due to its ability to regulate the APP proteolysis through the final biochemical step to produce Aβ, NCT can modify the balance between the detrimental and non-detrimental proteoforms. NCT is also reported to regulate the synaptic function by modulating the short-term and long-term synaptic plasticity [16]. Additionally, AD is considered to begin with organizational deficits that contributes to molecular progression of disease pathology at individual synapses [12, 13, 17]. Despite the emerging evidence on the importance of NCT in regulating synaptic plasticity and in the pathogenesis of AD, the nanoscale heterogeneity of NCT at synapses remains vague. Here, with the aid of ensemble-based nanoscopic imaging and analysis, we investigated the subsynaptic organization of NCT in functional zones of excitatory synapses and on the neuronal processes at nanoscale. Moreover, we also demonstrate the nanoorganization of NCT with APP and PS1, the catalytic subunit of γ-secretase, confirming the existence of their differential molecular aggregation at functional zones of the excitatory synapses. Additionally, we observed a subset of NCT domains independent of PS1, illustrating potentially unexplored regulatory mechanisms where the integral components of γ-secretase could work independent of each other.

## Results and discussion

**Fig. 1:**
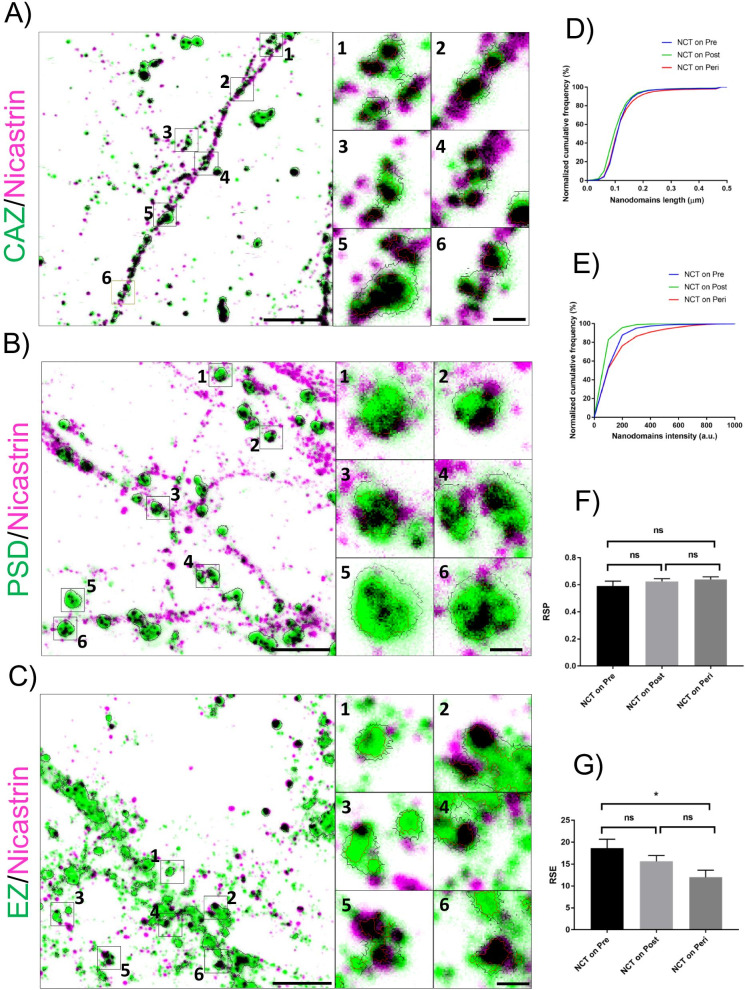
Subsynaptic compartmentalization of Nicastrin within functional zones of an excitatory synapse using STED microscopy. **A**–**C** Nanoscale distribution of Nicastrin (magenta) in pre/post/perisynapse with pseudocolour overlay of a presynaptic marker for the cytomatrix at the active zone (CAZ) in **A**, a postsynaptic marker for postsynaptic density (PSD) in **B** and a perisynaptic marker for the endocytic zone (EZ) in **C** i.e., Bassoon, Shank2 and Dynamin (green), respectively. Overlap between functional zones of the synapse and Nicastrin is presented in black. (1, 2, 3, 4, 5, 6) are magnified insets of regions indicated in **A**–**C**. Scale bar in **A**–**C** indicates 2.25 μm (left) and 300 nm (right, inset). **D**, **E** Indicate the distribution of the length **D** and intensity **E **of Nicastrin nanodomains in pre/post/perisynaptic compartments. **F**, **G** Comparison of RSP **F** and RSE **G** for quantifying colocalization of Nicastrin for functional zones (pre/CAZ, post/PSD, peri/EZ) of an excitatory synapse. The data are represented as mean ± SEM. Significance was determined by one-way analysis of variance (ANOVA) test followed by Tukey’s multiple comparison test. Indications of significance correspond to P values *P ≤ 0.05, **P ≤ 0.01, and ***P ≤ 0.001, ns P > 0.05. n = 5155 (pre), 3016 (post) and 3966 puncta (peri) from 3–4 biological repeats

To comprehend the molecular distribution of NCT within different functional zones of an excitatory synapse, we evaluated the nanoscale spatial heterogeneity of NCT by comparing its association with a presynaptic marker for the cytomatrix at the active zone (CAZ), a postsynaptic marker for postsynaptic density (PSD) and a perisynaptic marker for the endocytic zone (EZ). The distribution profile of NCT within different functional zones of the synapse was assessed by multi-colour ensemble-based super resolution imaging using Stimulated Emission Depletion microscopy (STED) and Airyscan super resolution microscopy (Fig. 1 and Additional file 1: Fig.S1). Using Airyscan super resolution microscopy, we observed a similar localization profile of NCT when evaluated against different markers for CAZ, namely Bassoon and Piccolo as well as against different markers of PSD, namely Shank2 and PSD95 (Additional file 1: Fig. S1A–F). A detailed workflow for the segmentation paradigm used to perform morpho-functional characterization of functional zones of an excitatory synapse is summarized in Supplementary information (Additional file 1: Materials and methods). We then evaluated the nanoscopic association of NCT within functional zones of the synapse using Bassoon (CAZ), Shank2 (PSD) and Dynamin (EZ) as synaptic reference markers using STED microscopy. NCT molecular domains (nanodomains) namely, nanodomain_NCT/pre_, nanodomain_NCT/post_ and nanodomain_NCT/peri_ indicate NCT nanodomains in regions marked positive for CAZ/PSD/EZ functional zones of the synapse (Fig. 1A–C). A gallery of representative STED images with synaptic markers and the associated nanodomains of NCT is presented (Fig. 1A–C). The morphological (length) and biophysical (intensity) traits of NCT nanodomains in pre/post/perisynapse were quantified (Fig. 1D, E). The distribution profile of length and intensity of nanodomain_NCT/pre_, nanodomain_NCT/post_ and nanodomain_NCT/peri_ is presented (Fig. 1D, E).

The length of nanodomain_NCT/post_ was significantly lower compared to both nanodomain_NCT/pre_ and nanodomain_NCT/peri_. On the other hand, the length of nanodomain_NCT/pre_ was similar to that of nanodomain_NCT/peri_ (Additional file 1: Fig. S2A, B). The intensity of NCT nanodomains also showed a similar pattern with nanodomain_NCT/post_ being significantly lower than that of nanodomain_NCT/pre_ and nanodomain_NCT/peri_, while the intensity of nanodomain_NCT/pre_ was similar to that of nanodomain_NCT/peri_ (Additional file 1: Fig. S2A, B). The length and intensity of the nanodomains of NCT present in neuronal processes (Additional file 1: Fig. S2C, D) and in synaptic subcompartments (Fig. 1D, E and Additional file 1: Fig. S2A, B) are summarized in (Additional file 1: Table S1). Our observations confirm that NCT, which is an integral component of the γ-secretase, is distributed into nanoscale domains within sub-diffraction sized functional zones of the synapses. The morphological attributes of the NCT nanodomains are comparable to that of PS1 nanodomains reported previously [13]. Our observations thus confirm that γ-secretase is organized into nanodomains within neuronal sub compartments. Next, we found that the colocalization of NCT in CAZ/PSD/EZ did not differ significantly between each other when measured by average spatial overlap across regions using Resolution Scaled Pearson’s (RSP) coefficient (Fig. 1F, G). On the contrary, the overall variability in fluorescence intensity across individual pixels in CAZ/PSD/EZ quantified by Resolution Scaled Error (RSE) between NCT on presynapse and perisynapse was significantly different, except when these zones were compared to the postsynapse (Fig. 1F, G). These observations of ensemble heterogeneity confirm the spatial variability of molecular organization of NCT, as revealed by nanodomain analysis. Additionally, NCT was also observed in inhibitory neurons (Additional file 1: Fig. S3A, B) and was associated with Gephyrin, a marker for inhibitory postsynapse on the hippocampal pyramidal neurons (Additional file 1: Fig. S3C).

The proteolytic processing of APP is deemed to occur by sequential cleavage, where the final snip is made by the γ-secretase [11]. Therefore, it was imperative to understand the nanoscopic association of NCT with APP as well as with the catalytic subunit, PS1. To quantify this association, we analyzed NCT regions marked positive for the presence of either APP or PS1. A gallery of STED images with PS1 and APP and the associated nanodomains of NCT is presented (Additional file 1: Fig. S4A, B). The nanodomain_NCT_ associated with PS1 and APP are referred to as nanodomain_NCT/PS_ and nanodomain_NCT/APP_, respectively. The distribution of length and intensity of nanodomain_NCT/PS_ and nanodomain_NCT/APP_ is shown (Additional file 1: Fig. S5A, B). Our results show a significantly higher length and intensity for nanodomain_NCT/APP_, as compared to nanodomain_NCT/PS_ (Additional file 1: Fig. S5C, D). Further, we found a significantly higher colocalization of NCT with PS1 compared to APP. In contrast, the variability of the quantified parameters of NCT with PS1 and with APP was similar (Additional file 1: Fig. S5E, F). In addition to the localization of NCT with PS1 indicating a functional γ-secretase, we also observed a subset of NCT nanodomains with variable intensities not localized to the proximity of PS1 (Additional file 1: Fig.S4A) and APP (Additional file 1: Fig. S4B). These observations are consistent with previous evidences on regulatory roles of NCT, independent of PS1 and γ-secretase complex [18]. It remains unclear if the individual components of γ-secretase could be transported independently to subcellular compartments, but it highlights novel unexplored mechanisms where these subunits could play an independent regulatory, anchoring or signaling role.

In the last decade, there has been a remarkable improvement in understanding the role of synaptic molecular organization in health and disease [12–15, 17, 19]. γ-secretase is involved in proteolysis of several substrate molecules and interact with molecules involved in synaptic transmission and plasticity [20]. We confirm that subunits of γ-secretase organize into discrete nanodomains on neuronal processes and within functional domains of excitatory synapses. Additionally, we observe that NCT and PS1 also form nanodomains independent of each other, where their functions could be independent of the γ-secretase activity. These observations of nanodomains of NCT and associated molecules in amyloidogenic processing highlight the role of multiprotein complexes in the proteolytic processing of APP. Our results are in resonance with the observation of the generation of Aβ in megadalton sized protein complexes isolated biochemically [21]. Though we attribute the proteolysis of APP by secretases as diffusional collisions, there is very little understanding on how the molecular trapping of substrates and secretases can result in the differential generation of proteoforms. These observations provide insight on how the molecular organization of individual components of multiprotein complexes can modulate synaptic imbalance and heterogeneity, leading to spatial differences in the breakdown of APP and other enzyme substrates at individual excitatory synapses. In addition to its canonical role as a component of γ-secretases, NCT is also involved in the regulation of short- and long-term plasticity [16, 22], further confirming its role as a synaptic molecule whose localization, regulation and function needs to be explored in depth.

## Supplementary Information

**Additional file 1: Table S1:** Summary of the quantitative estimation of morphological and biophysical properties of the different nanodomains of Nicastrin obtained using STED microscopy. **Figure S1.** **Distribution of Nicastrin within functional zones of an excitatory synapse using Airyscan super resolution microscopy**. **A**, **C** Indicate the pseudocolour coded distribution of the post- (Shank2 and PSD95) and pre-synaptic markers (Bassoon and Piccolo). **B**, **D** Indicate the pseudocolour coded distribution of Nicastrin and overlay with corresponding markers for functional zones of the synapse. The pseudocolour overlay of Nicastrin (green) with the postsynaptic marker Shank2 in red and the presynaptic marker Bassoon in blue is shown in **B**. The pseudocolour overlay of Nicastrin (green) with the postsynaptic marker PSD95 in red and the presynaptic marker Piccolo in blue is shown in **D**. **E** Magnified view of the boxed regions from pseudocolour overlay in **B** and **D**. Scale bar in **B**, **D** indicate 15 μm and in **E **2 μm. **F** Represents line scans connecting the centroids of pre- and post-synaptic reference molecules, indicating the distribution of Nicastrin with Shank2, Bassoon and PSD95, Piccolo. The X-and Y-axis represent the length (μm) and normalized intensity (a.u.) respectively. **Figure S2.** **Quantification of the nanoscale architecture of Nicastrin clusters within different functional zones of a synapse and on neuronal processes using STED microscopy**. **A**, **B** Diversity in Nicastrin (median/IQR 25–75% interval) clusters with respect to nanodomain length **A** and intensity **B** in pre/post/perisynapse. Significance was determined by Kruskal-Wallis test followed by Dunn’s multiple comparison test. Indications of significance correspond to P values *P ≤ 0.05, **P ≤ 0.01,and ***P ≤ 0.001, ns P > 0.05. n = 5155 (pre), 3016 (post) and 3966 (peri) puncta from 3-4 biological repeats. **C**, **D** Indicate the nanoscale architecture of Nicastrin clusters on the neuronal processes. The distribution of the length of Nicastrin nanodomains is shown in **C** and the intensity in **D**. n = 3521nanodomains from 3-4 biological repeats. **Figure S3.** **Distribution of Nicastrin in inhibitory neurons and within inhibitory synapses of pyramidal neurons using confocal microscopy**. **A** Evaluation of the presence of Nicastrin in Parvalbumin expressing GABAergic inhibitory neurons. The pseudocolour overlay represents Nicastrin in green and parvalbuminin red. The white arrows indicate the presence of both Nicastrin and parvalbumin, while the red arrows indicate the presence of Nicastrin in the absence of parvalbumin. Scale bar in **A** indicate 28 μm (upper panel) and 11 μm (lowerpanel). **B** Evaluation of the presence of Nicastrin in Parvalbumin/Calbindin expressing GABAergic inhibitory neurons. The pseudocolour overlay represents Nicastrin in green, parvalbumin in red and Calbindin in blue. Scale bar in **B** indicate 18 μm. **C** Indicate the distribution of Nicastrin within inhibitory synapses of pyramidal neurons. The pseudocolour overlay indicates Nicastrin ingreen and Gephyrin, a marker for the inhibitory postsynapses in magenta. The blue arrows indicate the clusters of Nicastrin overlapping with Gephyrin, while the yellow arrows indicate their independent distribution. Scale bar in **C** indicate 11 μm (left) and 2 μm (right, inset). **Figure S4.** **Discrete nanoscale association of Nicastrin with PS1 and APP on neuronal processes using STED microscopy**. **A** STED image of Nicastrin (magenta) with pseudocolour overlay of PS1 (green). (1, 2, 3) are magnifiedinsets of regions indicated in **A**. **B** STED image of Nicastrin (magenta) withpseudocolour overlay of APP (green). (1, 2, 3) are magnified insets of regions indicated in **B**. Scale bar in **A**, **B** indicates 3 μm (left) and 750 nm (right).The black contours represent segmented regions marking the continuous stretc.hof neuronal processes, marking the presence of Nicastrin. **Figure S5.** **Quantification of the nanoscale architecture of Nicastrin clusters associating with PS1 and APP on neuronal processes using STED microscopy**. **A**, **B** Indicate the distribution of the length **A** and intensity **B** of Nicastrin nanodomains with PS1 and APP. **C**, **D** Diversity in nanodomain length **C **and intensity **D** of Nicastrin clusters (median/IQR25–75% interval) associating with PS1 and APP. Significance was determined by unpaired two-tailed Mann-Whitney test. **E**, **F** Comparison of RSP **E** and RSE** F** for quantifying colocalization of Nicastrin with respect to PS1 and APP. The data are represented as mean ± SEM. Significance was determined by two-tailed unpaired Student’s t-test with Welch’s correction. Indications of significance correspond to P values *P ≤0.05, **P ≤ 0.01, and ***P ≤ 0.001, ns P > 0.05. n = 5417 (Nicastrin on PS1) and 7163 (Nicastrinon APP) puncta from 3-4 biological repeats. **Figure S6.** **Identification of synapse associated endocytic zone**.** A**, **B The workflow to detect synapse associated Dynamin (endocytic zones) using a combination of both super resolution (STED) and conventional (confocal) imaging paradigms**. **A** The confocal image of PSD marker and STED image of Dynamin were selected and thresholded to detect regions with high molecular content. They were then size filtered and obtained regions were converted into binary images. **B** The overlay of the masks confirms the overlap of segmented clusters of Dynamin with PSD. The automated evaluation of synaptic masks in the Dynamin positive domains was performed. Absence of Dynamin in PSD positive regions (green regions) were considered as negative, presence of both markers as positive (red regions) and dynamin alone as false positive (magenta regions). The paradigm involves sequential segmentation protocols to isolate Dynamin that is associated with the synaptic compartments and marking these segmented regions as synaptic endocytic zones. Scale bar in **A**, **B** indicate 5 μm. (Please refer to Supplementary information (Additional file 1: Materials and methods)) **Figure S7. Identification of Dynamin associated with Clathrin.** **A** Pseudocolour overlay of super resolved images of Clathrin (green) associated with Dynamin (Magenta) obtained using STED microscopy. **B** The workflow on super resolution images (STED) to detect Dynamin clusters colocalized with Clathrin, another marker for endocytic zone. The STED image of endocytic markers namely, Clathrin and Dynamin were selected and thresholded to detect regions with high molecular content. They were then size filtered and obtained regions were converted into binary images. The overlay of the masks confirms overlap of segmented clusters of Clathrin with Dynamin. Automated evaluation of Clathrin in the Dynamin positive domains was performed. For this purpose, the automatically detected Dynamin positive regions were transferred to the binary masks positive for Clathrin. Absence of Clathrin signal in Dynamin positive region was considered as negative, and those regions were not considered as endocytic regions. This segmentation protocol selectively evaluates domains enriched in both Clathrin and Dynamin, and therefore the presence of functional endocytic machinery. Scale bar in **A** indicate 5 μm and in **B** 2.25 μm. (Please Refer to Supplementary information (Additional file 1: Materials and methods)).

## Data Availability

The datasets collected and analysed during the current study is available from the corresponding author on reasonable request.

## Abbreviations

PS1: Presenilin 1
NCT: Nicastrin
APH1: Anterior Pharynx Defective 1
PEN2: Presenilin Enhancer 2
APP: Amyloid Precursor Protein
Aβ: Amyloid beta
AD: Alzheimer’s Disease
CAZ: Cytomatrix at the Active Zone
PSD: Post Synaptic Density
EZ: Endocytic Zone
STED: Stimulated Emission Depletion microscopy
