# Supplementary material for: Nanoscale organization of Nicastrin, the substrate receptor of the γ-secretase complex, as independent molecular domains
Source: Mol Brain. 2021 Oct 13;14:158. doi: 10.1186/s13041-021-00855-x (PMC8515736; doi:10.1186/s13041-021-00855-x)

**Additional File**

**Nanoscale organization of Nicastrin, the Substrate Receptor of the γ-Secretase Complex, as Independent Molecular Domains**

Shekhar Kedia^1^, Kousik Mandal^1^, Pallavi Rao Netrakanti^1^, Mini Jose^1^, Sangram S Sisodia^2^, Deepak Nair^1^

^1^Centre for Neuroscience, Indian Institute of Science, Bangalore 560012 (India).

^2^Centre for Molecular Neurobiology, Department of Neurobiology, The University of Chicago, IL 60637 (USA).

**Table**

**Table S1:** Summary of the quantitative estimation of morphological and biophysical properties of the different nanodomains of Nicastrin obtained using STED microscopy.

| **Category/Parameter** | **Length (µm)** | **Intensity (a.u.)** | **Normalized intensity** |
| --- | --- | --- | --- |
| **Nanodomain**_N_**_CT_** | 0.121+/-0.000  (0.110, 0.090-0.136) | 135.70+/-1.74  (110.00, 66.96-171.90) | 1.00+/-0.012  (0.810, 0.493-1.266) |
| **Nanodomain**_N_**_CT/Pre_** | 0.128+/-0.001  (0.116, 0.096-0.142) | 167.70+/-2.58  (142.30,103.60-194.50) | 1.235+/-0.021  (1.048,0.763-1.433) |
| **Nanodomain**_N_**_CT/Post_** | 0.117+/-0.001  (0.106, 0.084-0.134) | 77.37+/-1.89  (58.50, 40.35-90.05) | 0.570+/-0.013  (0.431, 0.297-0.663) |
| **Nanodomain**_N_**_CT/Peri_** | 0.132+/-0.001  (0.115, 0.094-0.146) | 201.60+/-4.30  (141.50, 93.18-234.80) | 1.485+/-0.031  (1.042, 0.686-1.730) |
| **Nanodomain**_N_**_CT/PS_** | 0.139+/-0.001  (0.123, 0.101-0.153) | 229.10+/-3.89  (167.50, 112.30-292.20) | 1.688+/-0.028  (1.234, 0.827-2.153) |
| **Nanodomain**_N_**_CT/APP_** | 0.155+/-0.002  (0.132, 0.104-0.172) | 281.20+/-5.31  (230.40, 149.90-358.20) | 2.072+/-0.039  (1.697, 1.104-2.639) |

The values indicated are mean+/-SEM, while values in brackets represent the median, IQR from 25% percentile to 75% percentile. Normalized intensity is normalized with respect to the mean of the global NCT nanodomain intensity for nanodomain_NCT_.

**Figure Legends**

**Figure S1. Distribution of Nicastrin within functional zones of an excitatory synapse using Airyscan super resolution microscopy.** (A, C) Indicate the pseudocolour coded distribution of the post- (Shank2 and PSD95) and pre-synaptic markers (Bassoon and Piccolo). (B, D) Indicate the pseudocolour coded distribution of Nicastrin and overlay with corresponding markers for functional zones of the synapse. The pseudocolour overlay of Nicastrin (green) with the postsynaptic marker Shank2 in red and the presynaptic marker Bassoon in blue is shown in (B). The pseudocolour overlay of Nicastrin (green) with the postsynaptic marker PSD95 in red and the presynaptic marker Piccolo in blue is shown in (D). (E) Magnified view of the boxed regions from pseudocolour overlay in (B) and (D). Scale bar in (B, D) indicate 15 μm and in (E) 2 μm. (F) Represents line scans connecting the centroids of pre- and post-synaptic reference molecules, indicating the distribution of Nicastrin with Shank2, Bassoon and PSD95, Piccolo. The X- and Y-axis represent the length (μm) and normalized intensity (a.u.) respectively.

**Figure S2. Quantification of the nanoscale architecture of Nicastrin clusters within different functional zones of a synapse and on neuronal processes using STED microscopy.** (A, B) Diversity in Nicastrin (median/IQR 25%-75% interval) clusters with respect to nanodomain length (A) and intensity (B) in pre/post/perisynapse. Significance was determined by Kruskal-Wallis test followed by Dunn’s multiple comparison test. Indications of significance correspond to P values *P ≤ 0.05, **P ≤ 0.01, and ***P ≤ 0.001, ns P > 0.05. n = 5155 (pre), 3016 (post) and 3966 (peri) puncta from 3-4 biological repeats. (C, D) Indicate the nanoscale architecture of Nicastrin clusters on the neuronal processes. The distribution of the length of Nicastrin nanodomains is shown in (C) and the intensity in (D). n = 3521 nanodomains from 3-4 biological repeats.

**Figure S3. Distribution of Nicastrin in inhibitory neurons and within inhibitory synapses of pyramidal neurons using confocal microscopy.** (A) Evaluation of the presence of Nicastrin in Parvalbumin expressing GABAergic inhibitory neurons. The pseudocolour overlay represents Nicastrin in green and parvalbumin in red. The white arrows indicate the presence of both Nicastrin and parvalbumin, while the red arrows indicate the presence of Nicastrin in the absence of parvalbumin. Scale bar in (A) indicate 28 μm (upper panel) and 11 μm (lower panel). (B) Evaluation of the presence of Nicastrin in Parvalbumin/Calbindin expressing GABAergic inhibitory neurons. The pseudocolour overlay represents Nicastrin in green, parvalbumin in red and Calbindin in blue. Scale bar in (B) indicate 18 μm. (C) Indicate the distribution of Nicastrin within inhibitory synapses of pyramidal neurons. The pseudocolour overlay indicates Nicastrin in green and Gephyrin, a marker for the inhibitory postsynapses in magenta. The blue arrows indicate the clusters of Nicastrin overlapping with Gephyrin, while the yellow arrows indicate their independent distribution. Scale bar in (C) indicate 11 μm (left) and 2 μm (right, inset).

**Figure S4. Discrete nanoscale association of Nicastrin with PS1 and APP on neuronal processes using STED microscopy.** (A) STED image of Nicastrin (magenta) with pseudocolour overlay of PS1 (green). (1, 2, 3) are magnified insets of regions indicated in (A). (B) STED image of Nicastrin (magenta) with pseudocolour overlay of APP (green). (1, 2, 3) are magnified insets of regions indicated in (B). Scale bar in (A, B) indicates 3 μm (left) and 750 nm (right). The black contours represent segmented regions marking the continuous stretch of neuronal processes, marking the presence of Nicastrin.

**Figure S5. Quantification of the nanoscale architecture of Nicastrin clusters associating with PS1 and APP on neuronal processes using STED microscopy.** (A, B) Indicate the distribution of the length (A) and intensity (B) of Nicastrin nanodomains with PS1 and APP. (C, D) Diversity in nanodomain length (C) and intensity (D) of Nicastrin clusters (median/IQR 25%-75% interval) associating with PS1 and APP. Significance was determined by unpaired two-tailed Mann-Whitney test. (E, F) Comparison of RSP (E) and RSE (F) for quantifying colocalization of Nicastrin with respect to PS1 and APP. The data are represented as mean +/- SEM. Significance was determined by two-tailed unpaired Student’s t-test with Welch’s correction. Indications of significance correspond to P values *P ≤ 0.05, **P ≤ 0.01, and ***P ≤ 0.001, ns P > 0.05. n = 5417 (Nicastrin on PS1) and 7163 (Nicastrin on APP) puncta from 3-4 biological repeats.

**Figure S6. Identification of synapse associated endocytic zone.** (A, B) The workflow to detect synapse associated Dynamin (endocytic zones) using a combination of both super resolution (STED) and conventional (confocal) imaging paradigms. (A) The confocal image of PSD marker and STED image of Dynamin were selected and thresholded to detect regions with high molecular content. They were then size filtered and obtained regions were converted into binary images. (B) The overlay of the masks confirms the overlap of segmented clusters of Dynamin with PSD. The automated evaluation of synaptic masks in the Dynamin positive domains was performed. Absence of Dynamin in PSD positive regions (green regions) were considered as negative, presence of both markers as positive (red regions) and dynamin alone as false positive (magenta regions). The paradigm involves sequential segmentation protocols to isolate Dynamin that is associated with the synaptic compartments and marking these segmented regions as synaptic endocytic zones. Scale bar in (A, B) indicate 5 μm.

**Figure S7.** **Identification of Dynamin associated with Clathrin.** (A) Pseudocolour overlay of super resolved images of Clathrin (green) associated with Dynamin (Magenta) obtained using STED microscopy. (B) The workflow on super resolution images (STED) to detect Dynamin clusters colocalized with Clathrin, another marker for endocytic zone. The STED image of endocytic markers namely, Clathrin and Dynamin were selected and thresholded to detect regions with high molecular content. They were then size filtered and obtained regions were converted into binary images. The overlay of the masks confirms overlap of segmented clusters of Clathrin with Dynamin. Automated evaluation of Clathrin in the Dynamin positive domains was performed. For this purpose, the automatically detected Dynamin positive regions were transferred to the binary masks positive for Clathrin. Absence of Clathrin signal in Dynamin positive region was considered as negative, and those regions were not considered as endocytic regions. This segmentation protocol selectively evaluates domains enriched in both Clathrin and Dynamin, and therefore the presence of functional endocytic machinery. Scale bar in (A) indicate 5 μm and in (B) 2.25 μm.

**Materials and Methods**

**Experimental Animals**

Wild type Sprague-Dawley rats were obtained from the Institutional Central Animal Facility and were housed under pathogen free environment. The animals were maintained in a temperature-controlled room on 12 h light/12 h dark cycle with *ad libitum* access to food and water. All experiments involving animals were carried out in accordance with institutional guidelines for the use and care of animals after approval from the Institutional Animal Ethics Committee (IAEC), Indian Institute of Science, Bangalore, India.

**Primary Hippocampal Culture**

Mixed sex primary hippocampal neurons cultured from postnatal day 0 or 1 (P0-P1) wild type Sprague-Dawley rats were prepared and maintained according to a previously described protocol [1–4]. The cells were seeded at a density of 0.1×10^6^ cells/mL in 18 mm #1.5 (corrected for 0.17+/- 0.01) glass coverslips (coated with poly-D-lysine at a concentration of 100 μg/mL) in a 12-well cell culture plate. Primary hippocampal neurons were used for immunocytochemical evaluation at DIV 20-21.

**Antibodies**

The primary antibodies used in this study were anti-Nicastrin (1:500, NCT54) [5–7], anti-Bassoon (1:1000, Synaptic Systems, Cat# 141021), anti-Piccolo (1:500, Synaptic Systems, Cat#142104), anti-Shank2 (1:500, Synaptic Systems, Cat# 162204), anti-PSD95 (1:500, Thermo Scientific, Cat# MA1-046), anti-Dynamin (1:1000, Upstate/Millipore, Cat# 05-319) detects Dynamin1/2 (referred to as Dynamin), anti-Clathrin (1:500, Abcam, Cat# ab21679), anti-APP-CT (1:500, Biolegend/Covance, Cat# 802801) and anti-Presenilin1 (1:500, Merck Millipore, Cat# MAB5232), anti-Parvalbumin (1:500, Synaptic Systems, Cat# 195004), anti-Gephyrin (1:500, Synaptic Systems, Cat# 147111), anti-Calbindin (1:500, Sigma-Aldrich, Cat# C8666). The secondary antibodies used were Alexa Fluor 594 (1:400, Life Technologies, Cat# A11037), Alexa Fluor 647 (1:400, Life Technologies, Cat# A21450), Alexa Fluor 488 (1:400, Life Technologies, Cat# A11029), Abberior Star Red (1:400, Abberior, Cat# 2-0002-011-2) and Abberior Star Red (1:400, Abberior, Cat# 2-0112-011-8).

**Immunocytochemistry**

Immunocytochemistry was performed as described previously [1–3]. Cells were mounted with prolong (Molecular Probes, cat. no. MAN0010261). After incubation for 24 h in dark at room temperature, STED imaging was performed.

**Confocal Microscopy**

Immunocytochemical samples of primary hippocampal neurons co-labelled with markers for inhibitory neurons and inhibitory synapses with Nicastrin were imaged using confocal microscopy. Confocal images were acquired using a Leica SP8 microscope sampled at 92 nm/pixel for localization of Nicastrin with a marker for inhibitory synapses and/or at 185 nm/pixel or246 nm/pixel for Nicastrin with markers for inhibitory neurons, using a 63X objective with a numerical aperture of 1.4.

**Airyscan Super Resolution Microscopy**

Immunocytochemical samples of primary hippocampal neurons co-labelled with presynaptic and post synaptic markers with Nicastrin were used for super-resolution imaging by Airyscan microscopy. Airyscan was performed on Zeiss LSM 880 equipped with 32 array detectors for acquisition of super-resolution images. We then performed confocal and Airyscan imaging on non-overlapping regions of neuronal processes of the immunolabelled hippocampal neurons with an effective field size of 46.5 X46.5 μm^2^ sampled at 43 nm/pixel using a 63X objective with a numerical aperture of 1.4. For image acquisition, 488, 543 and 633 nm lasers were used. The 100 nm Tetraspeck (Invitrogen, cat. no. T7279) beads emitting in multiple wavelengths were used as a resolution standard, providing a full width at half maxima of 110-130 nm across the acquisition channels. The illumination intensities, sampling of the images, digital and analogue gain of the detectors, emission window for each fluorescent channel and their corresponding pinhole sizes were maintained constant across acquisition. The raw images acquired using Airyscan mode were processed using Zeiss acquisition and analysis software of the microscope to generate final super resolution images. The reconstruction parameters were kept constant throughout the samples.

**Stimulated Emission Depletion Microscopy (STED)**

A commercial STED inverted microscope (Abberior Expert Line 775 nm, Abberior Instruments GmbH, Göttingen, Germany) was used to obtain super resolved images of the same region with a sampling of 15 nm by following a previously described protocol [1–3]. The STED system was equipped with a pulsed depletion laser at 775 nm and two pulsed excitation lasers at 561 nm and 640 nm. The power of the lasers was adjusted to 70%, 50% and 40% of their respective total power for 561 nm, 640 nm and 775 nm, respectively [1–3].

**Morpho-functional Characterization of Endocytic Zone of an Excitatory Synapse**

To verify the localization of Dynamin1/2 (referred to as Dynamin) in the synaptic compartment, we compared both super resolution and confocal microscopy on different functional markers of the synapse. For this, the primary hippocampal neurons were counter labelled with two postsynaptic markers (data not shown), with a postsynaptic marker and Dynamin (Additional File 1, Fig. S6) or with two endocytic markers (Dynamin with Clathrin) (Additional File, Fig. S7). When using confocal microscopy, markers of the same functional zones showed high overlap, while markers of different functional zones displayed partial overlap. Super resolved imaging of the same exhibited high colocalization between two markers of the same synaptic functional zone (overlap in >95% of pixel area). Colocalization was mutually exclusive between different functional zones marked by postsynaptic density markers and Dynamin (overlap in <15% of pixel area), consistent with previous reports. The labelling of PSD markers and Dynamin were juxtaposed to each other, accounting for this minor overlap and confirming that these functional zones can be resolved using STED microscopy. Similar results were obtained on assessing the localization of Dynamin against active zone markers, confirming that Dynamin is excluded from both Cytomatrix of the active zone and the postsynaptic density. In both cases, our reports were consistent with previous evidences of the same using super resolution strategies [2,8–10].

The next step involved identification of Dynamin associated with synapses and marking them as endocytic zones. This paradigm involved sequential segmentation protocols to isolate Dynamin associated with the synaptic compartments and marking these segmented regions as synaptic endocytic zones (Additional File 1, Fig. S6, S7). The conventional microscopy images of synaptic markers obtained by confocal microscopy overlapped partially with the super resolved images of Dynamin (STED) from the same regions. This partial overlap between synaptic markers and Dynamin was used to localize synapse associated endocytic zones. An automated workflow was generated to segment out both the synaptic compartment observed by confocal microscopy and Dynamin labelling observed by super resolution imaging (Additional File 1, Fig. S6, S7). Using MetaMorph image analysis software (Molecular Devices), global intensity threshold was performed using the function “Auto threshold for light objects” for images obtained for each marker. The images were thresholded using a minimum cut off calculated by the sum of average intensity and standard deviation obtained from the previous step to detect synaptic clusters or neuronal compartments enriched with Dynamin. This was followed by use of “Integrated Morphometry analysis” (IMA) to select all the synaptic clusters and Dynamin domains which were greater than 500 Pixel area. The filtered objects were transformed into a binary image which represented an independent mask of the synaptic regions and Dynamin enriched compartments. An overlay of the masks exhibited a high overlap between synaptic markers and super resolved Dynamin. To understand the amount of ‘false positives’ (regions enriched in non-synaptic Dynamin), we evaluated the percentage of detected Dynamin positive regions associated with synaptic compartments and found this to be greater than 96%. This confirmed with high probability that the filtered Dynamin regions were indeed associated with synaptic substructures, and thus were labelled as endocytic zones. Additionally, to confirm the presence of functional endocytic machinery in these endocytic zones, a similar analysis was performed on the super resolved images of Dynamin colocalized to Clathrin, another marker for endocytic Zone (Additional File 1, Fig. S7). With a similar workflow that was used previously, we performed analysis with a pixel area cut off of 500- and 100-pixel area. With a 500-pixel area cut off, we had 100% of Dynamin enriched regions overlapping with Clathrin, while with 100-pixel area cut off this was reduced to 98%, confirming that the Dynamin enriched compartments marked as endocytic zones (Additional File 1, Fig. S7) fulfilled both spatial and functional criteria that classify them as sites of active endocytosis.

**Semiautomated Detection of Functional Zones of an Excitatory Synapse**

The synapses were identified from the rest of the neuronal processes following a protocol described earlier using an IMA plugin running within the MetaMorph software (Molecular Devices) [1–3]. A semiautomated segmentation procedure was employed to execute nanoscale morphometry analysis. An object-based strategy was used for segmenting images into several objects of interest. The analysis on STED super resolved images was used to distinguish functional zones of excitatory synapses from the rest of the neuronal processes that correspond to CAZ/PSD functional zones. The intensity of the STED images of markers for different functional zones of the synapse was thresholded to generate a mask of the puncta and these puncta were segmented based on the thresholding. These masks were then analysed and were filtered through the IMA module for synaptic puncta using several morphological filters such as length, breadth and area which characterizes the synapse morphometry [1–3,11,12].

**Resolution Scaled Pearson’s Coefficient (RSP) and Resolution Scaled Error (RSE) Analysis**

Resolution Scaled Pearson’s Coefficient (RSP) and Resolution Scaled Error (RSE) were used to understand pixel by pixel correlation between two images and to extract the local variability of recorded signals between pixels. Across super resolved images of similar resolution, RSP evaluates the spatial similarity between the data. A high correlation corresponds to homogeneity between signals at nanoscale, whereas a lower correlation suggested an inhomogeneity which can be a result of spatial dissociation of signals at nanoscale. RSE represents the variability of intensity between both images. Higher values of RSE indicates an inhomogeneity in the brightness and contrast. Larger the RSE, lower the correlated changes in intensity across the images in a region of interest. Together these values represent variability in localization in a region of interest. However, these values do not provide information on the nanoscale aggregation properties between the compared images. RSP and RSE data was obtained using NanoJ-SQUIRREL, a plugin supported by Fiji as described earlier [1,2]. The reference image was either a marker for a functional compartment of the synapse (pre/post/peri) or APP or PS1, for assessing the association of NCT with these zones [1,2]. The subject image was that of NCT. Both reference and subject images were STED images. The representative resolution scaling function was calculated automatically through optimization [1,2]. The RSP and RSE values for each image was calculated and plotted.

**Super Resolution Cluster Analysis**

With the aid of semiautomated detection of functional zones of an excitatory synapse, the synapses (CAZ/PSD/EZ) were identified and filtered. Functional zones of the synapse were detected from the STED super resolved images of the synaptic markers (reference image). The reference image was either a marker for a functional compartment of the synapse (pre/post/peri) or APP or PS1, for assessing the association of NCT with these zones [1–3]. The STED super resolved images (subject image) of the proteins of interest (NCT) were analysed to quantify clusters of molecular aggregation (NCT nanodomains). Nanodomains were identified from STED images by a custom algorithm written as a plug-in supported by MetaMorph (Molecular Devices) [1–3]. NCT Nanodomains that were localized to different functional zones of the synapse or those associated with APP or PS1 were identified from STED images by PalmTracer plugin [1–3]. Nanodomains were quantified by a bi-dimensional Gaussian fitting using a previously described protocol [1–3]. The morphological and biophysical traits of NCT nanodomains such as length and intensity of the nanodomains were quantified for each category. For the measurement of nanodomain length, SigmaY was computed and multiplied with 2.3 [1–3]. The Gaussian fitting was performed on each cluster that was detected as a nanodomain. As described previously, the nanodomains with length above 500 nm and intensity values above 5000 a.u. were excluded [1–3].

**Statistics**

Statistical analysis was performed using GraphPad Prism version 7.04 for Windows, GraphPad Software, La Jolla California USA, ([www.graphpad.com](http://www.graphpad.com/)). All statistical values are shown as either mean +/- SEM for RSP/RSE or median (IQR 25% to 75% interval) for nanodomains, unless otherwise indicated. RSP and RSE datasets were compared using two-tailed unpaired Student’s t-test with Welch’s correction for two-group or one-way analysis of variance (ANOVA) test followed by Tukey’s multiple comparison test for multi-group. Nanodomain datasets were tested by unpaired two-tailed Mann-Whitney test for two-group or Kruskal-Wallis test followed by Dunn’s multiple comparison test for multi-group. Indications of significance correspond to P values *P ≤ 0.05, **P ≤ 0.01, and ***P ≤ 0.001, ns P > 0.05. The calculation of the required sample sizes was obtained from the power and sample size calculator from statistical solutions [1,2,13]. To account for variability, data was obtained from 3-4 independent cultures.

**References**

1. Kedia S, Ramakrishna P, Netrakanti PR, Singh N, Sisodia SS, Jose M, et al. Alteration in synaptic nanoscale organization dictates amyloidogenic processing in Alzheimer’s disease. iScience; 2021;24:101924.

2. Kedia S, Ramakrishna P, Netrakanti PR, Jose M, Sibarita JB, Nadkarni S, et al. Real-time nanoscale organization of amyloid precursor protein. Nanoscale. 2020;12:8200–15.

3. Kedia S, Ramanan N, Nair D. Quantifying molecular aggregation by super resolution microscopy within an excitatory synapse from mouse hippocampal neurons. STAR Protoc. 2021;2:100470.

4. Beaudoin GMJ, Lee S-H, Singh D, Yuan Y, Ng Y-G, Reichardt LF, et al. Culturing pyramidal neurons from the early postnatal mouse hippocampus and cortex. Nat Protoc. 2012;7:1741–54.

5. Kim SH, Yin YI, Li YM, Sisodia SS. Evidence that assembly of an active γ-secretase complex occurs in the early compartments of the secretory pathway. J Biol Chem. 2004;279:48615–9.

6. Kim SH, Ikeuchi T, Yu C, Sisodia SS. Regulated hyperaccumulation of presenilin-1 and the “γ -secretase” complex: Evidence for differential intramembranous processing of transmembrane substrates. J Biol Chem. 2003;278:33992–4002.

7. Renzi F, Zhang X, Rice WJ, Torres-Arancivia C, Gomez-Llorente Y, Diaz R, et al. Structure of γ-secretase and its trimeric pre-activation intermediate by single-particle electron microscopy. J Biol Chem. 2011;286:21440–9.

8. Wilhelm BG, Mandad S, Truckenbrodt S, Kröhnert K, Schäfer C, Rammner B, et al. Composition of isolated synaptic boutons reveals the amounts of vesicle trafficking proteins. Science. 2014;344:1023–8.

9. Venkatesan S, Subramaniam S, Rajeev P, Chopra Y, Jose M, Nair D. Differential scaling of synaptic molecules within functional zones of an excitatory synapse during homeostatic plasticity. eNeuro. 2020;7:1–16.

10. Helm MS, Dankovich TM, Mandad S, Rammner B, Jähne S, Salimi V, et al. A large-scale nanoscopy and biochemistry analysis of postsynaptic dendritic spines. Nat Neurosci. 2021;24:1151–62.

11. Harris M, Stevens JK. Dendritic Spines of CA1 Pyramidal Cells in the Rat Hippocampus : Serial Electron Microscopy with Reference to Their Biophysical Characteristics. J Neurosci. 1989;9:2982–97.

12. Harris KM, Weinberg RJ. Ultrastructure of synapses in the mammalian brain. Cold Spring Harb Perspect Biol. 2012;4:7.

13. Nair D, Hosy E, Petersen JD, Constals A, Giannone G, Choquet D, et al. Super-resolution imaging reveals that AMPA receptors inside synapses are dynamically organized in nanodomains regulated by PSD95. J Neurosci. 2013;33:13204–24.

Figures

Figure S1


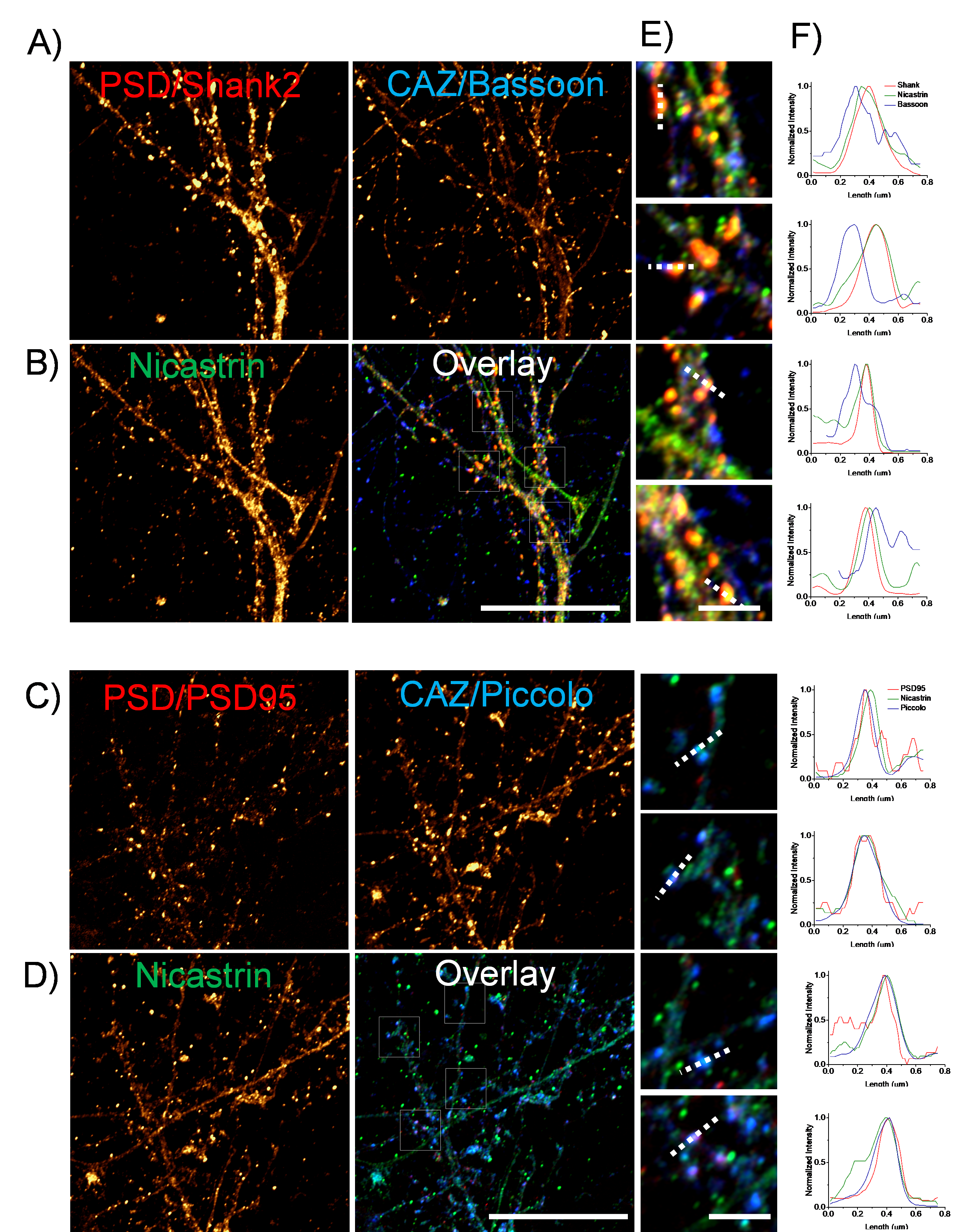


Figure S2


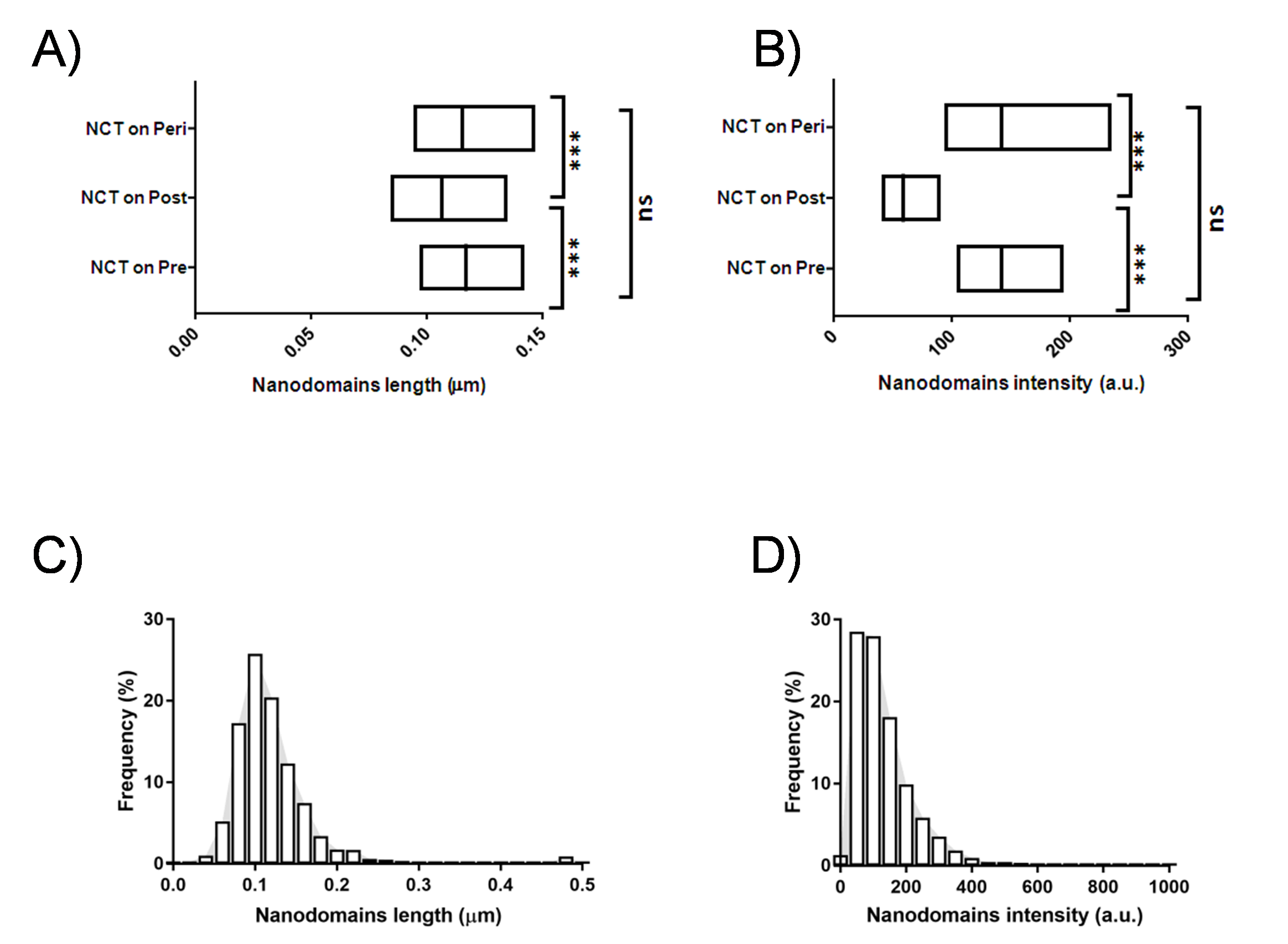


Figure S3


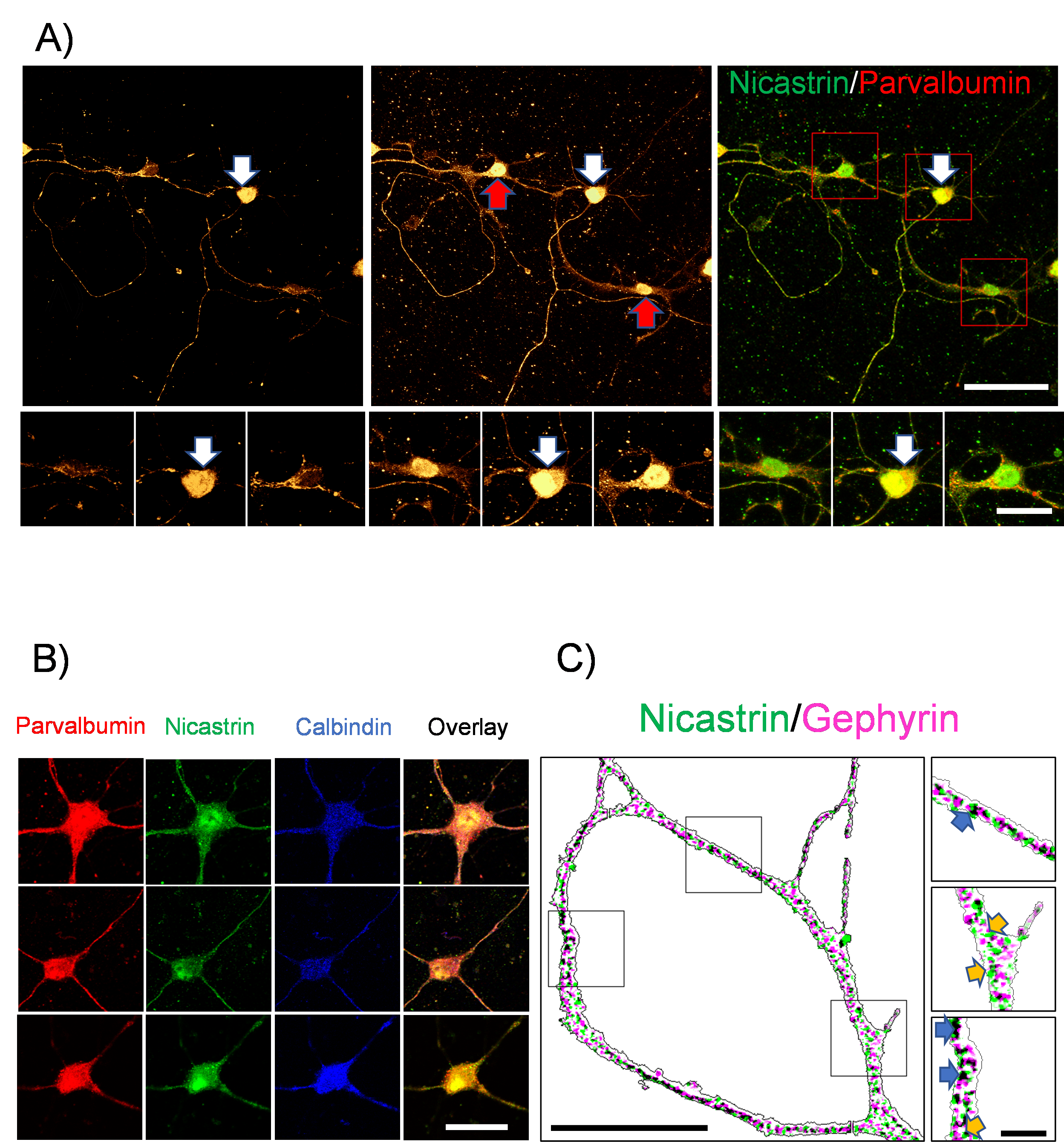


Figure S4


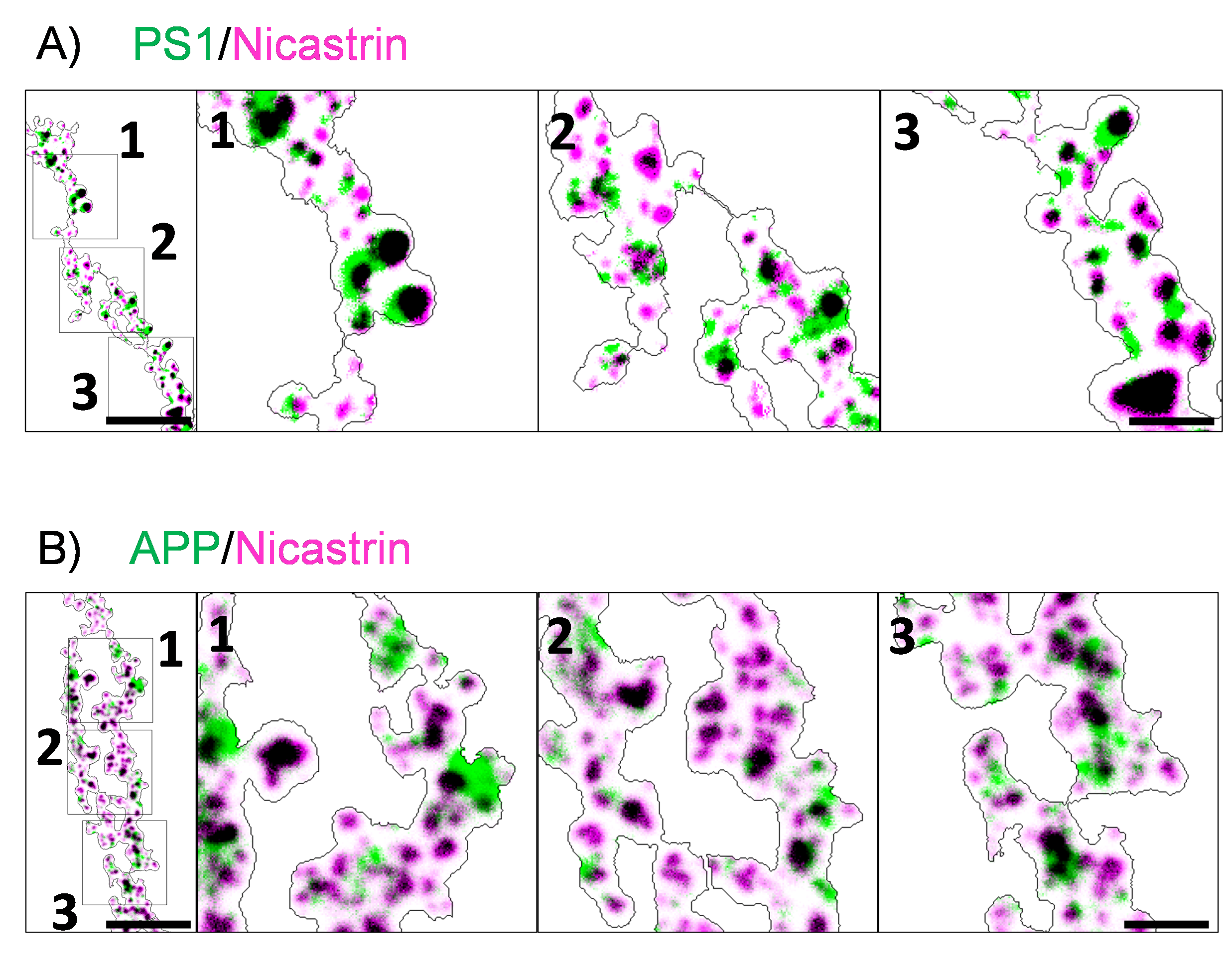


Figure S5


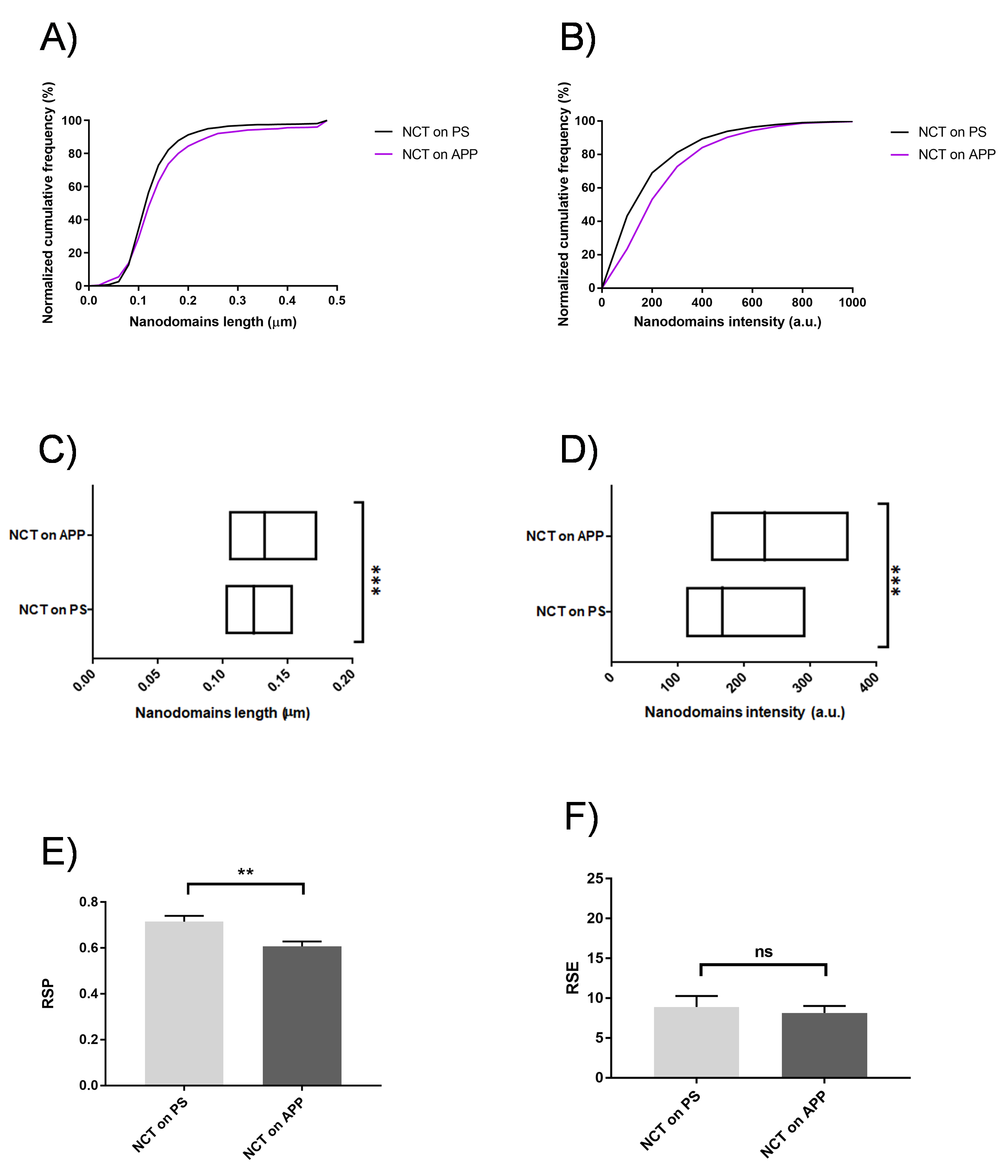


Figure S6


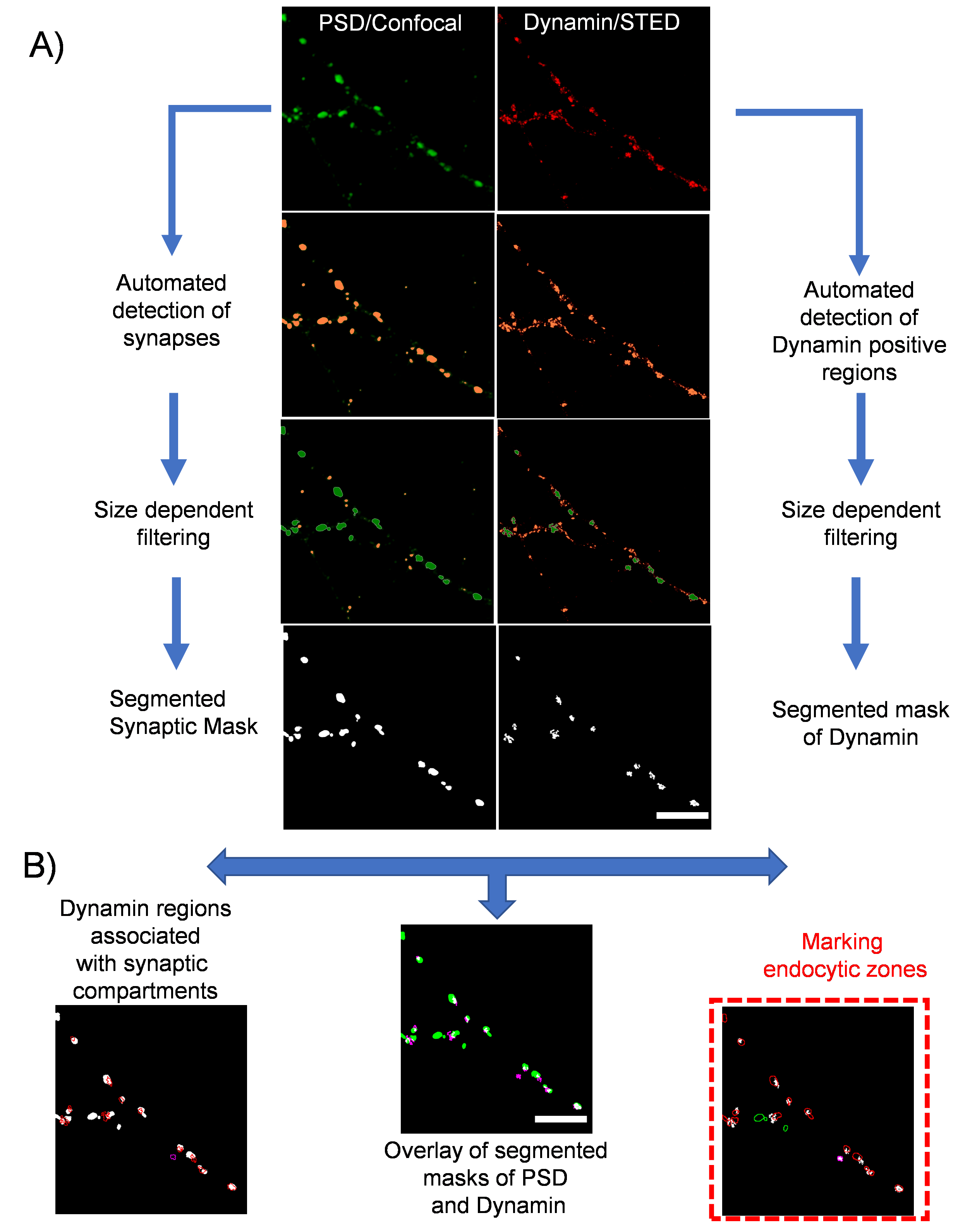


Figure S7


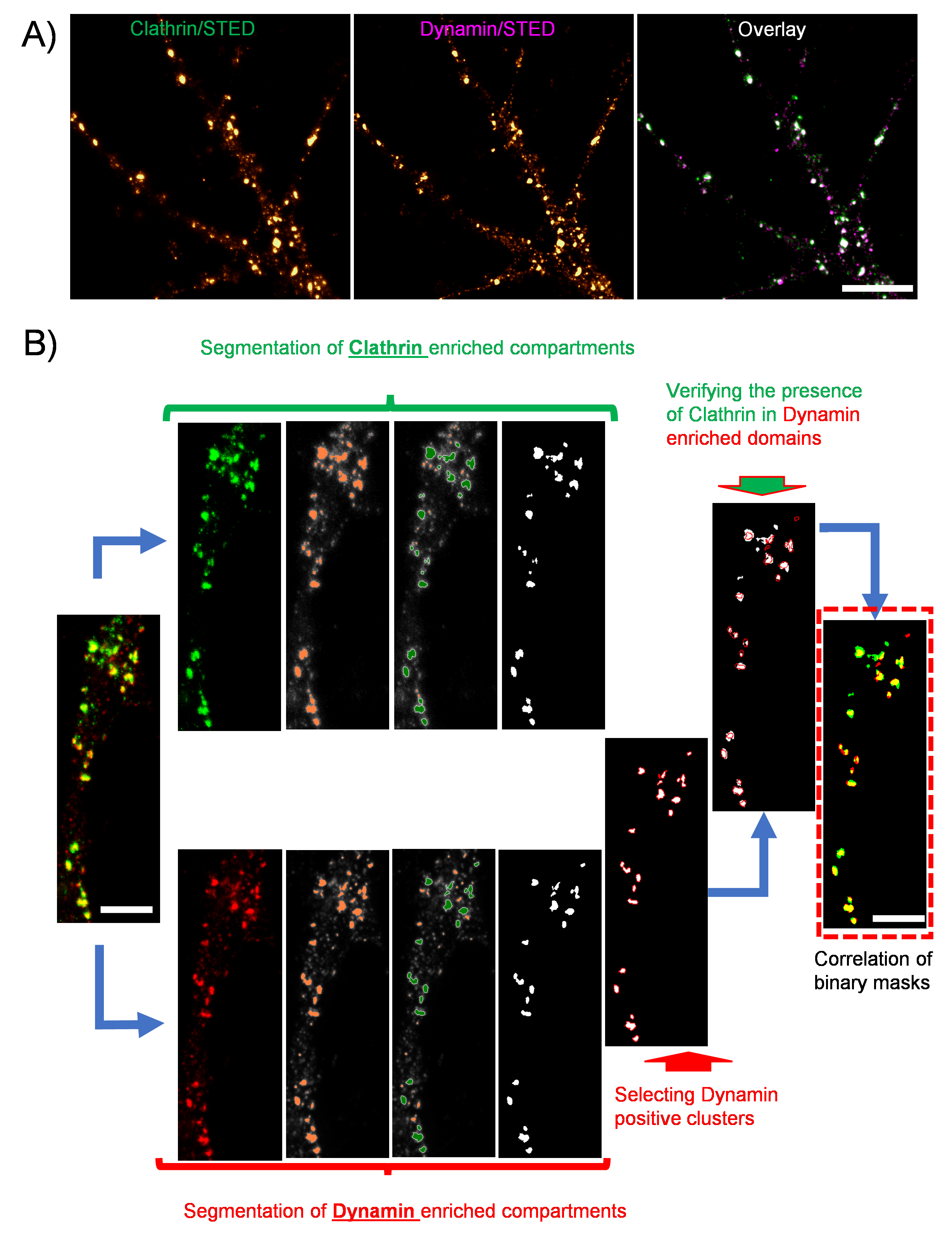

Supplement: Supplementary file 1 — Additional file 1: Table S1: Summary of the quantitative estimation of morphological and biophysical properties of the different nanodomains of Nicastrin obtained using STED microscopy. Figure S1. Distribution of Nicastrin within functional zones of an excitatory synapse using Airyscan super resolution microscopy. A, C Indicate the pseudocolour coded distribution of the post- (Shank2 and PSD95) and pre-synaptic markers (Bassoon and Piccolo). B, D Indicate the pseudocolour coded distribution of Nicastrin and overlay with corresponding markers for functional zones of the synapse. The pseudocolour overlay of Nicastrin (green) with the postsynaptic marker Shank2 in red and the presynaptic marker Bassoon in blue is shown in B. The pseudocolour overlay of Nicastrin (green) with the postsynaptic marker PSD95 in red and the presynaptic marker Piccolo in blue is shown in D. E Magnified view of the boxed regions from pseudocolour overlay in B and D. Scale bar in B, D indicate 15 μm and in E 2 μm. F Represents line scans connecting the centroids of pre- and post-synaptic reference molecules, indicating the distribution of Nicastrin with Shank2, Bassoon and PSD95, Piccolo. The X-and Y-axis represent the length (μm) and normalized intensity (a.u.) respectively. Figure S2. Quantification of the nanoscale architecture of Nicastrin clusters within different functional zones of a synapse and on neuronal processes using STED microscopy. A, B Diversity in Nicastrin (median/IQR 25–75% interval) clusters with respect to nanodomain length A and intensity B in pre/post/perisynapse. Significance was determined by Kruskal-Wallis test followed by Dunn’s multiple comparison test. Indications of significance correspond to P values *P ≤ 0.05, **P ≤ 0.01,and ***P ≤ 0.001, ns P > 0.05. n = 5155 (pre), 3016 (post) and 3966 (peri) puncta from 3-4 biological repeats. C, D Indicate the nanoscale architecture of Nicastrin clusters on the neuronal processes. The distribution of the length of Nicastrin [file 13041_2021_855_MOESM1_ESM.docx]
